# The roles of apoptotic pathways in the low recovery rate after cryopreservation of dissociated human embryonic stem cells

**DOI:** 10.1002/btpr.368

**Published:** 2010-05

**Authors:** Xia Xu, Sally Cowley, Christopher J Flaim, William James, Leonard Seymour, Zhanfeng Cui

**Affiliations:** 1 Dept. of Engineering Science, Institute of Biomedical Engineering, University of OxfordOxford, U.K.; 2 Sir William Dunn School of Pathology, University of OxfordOxford, U.K.; 3 Dept. of Clinical Pharmacology, University of OxfordOxford, U.K.

**Keywords:** cryopreservation of hES cells, apoptosis pathways, reactive oxygen species, F-actin, apoptosis inhibitors

## Abstract

Human embryonic stem (hES) cells have enormous potential for clinical applications. However, one major challenge is to achieve high cell recovery rate after cryopreservation. Understanding how the conventional cryopreservation protocol fails to protect the cells is a prerequisite for developing efficient and successful cryopreservation methods for hES cell lines and banks. We investigated how the stimuli from cryopreservation result in apoptosis, which causes the low cell recovery rate after cryopreservation. The level of reactive oxygen species (ROS) is significantly increased, F-actin content and distribution is altered, and caspase-8 and caspase-9 are activated after cryopreservation. p53 is also activated and translocated into nucleus. During cryopreservation apoptosis is induced by activation of both caspase-8 through the extrinsic pathway and caspase-9 through the intrinsic pathway. However, exactly how the extrinsic pathway is activated is still unclear and deserves further investigation. © 2010 American Institute of Chemical Engineers Biotechnol. Prog., 2010

## Introduction

hES cells have enormous potential for clinical applications, offering therapies for a wide range of degenerative diseases and disorders such as diabetes and Parkinson's disease.^1^ Long term storage or banking is a prerequisite for hES cell applications. For potential therapeutic or commercial applications, banking requiring a large amount of hES cells is required. Hence, slow freezing, rather than vitrification, is more practical. One of the current technical challenges is how to achieve high cell recovery rate after cryopreservation.

In cryopreservation, cryoprotectant agent (CPA), such as dimethylsulfoxide (DMSO), is added to cells to protect them during freezing. However, osmotic stress during CPA addition results in volume change of the cells. After CPA addition, the cells are then frozen at a slow freezing rate until −80°C. In this step, cells are exposed to cold stress and simultaneously experience osmotic shock again. Before use, the cells are rapidly thawed to minimize the risk of ice recrystallization, and CPA is removed. As in the addition step, the cell volume changes due to osmolarity differences between the intracellular and extracellular environment. Hence, in each step of cryopreservation, external stresses are introduced, which may lead to cell damage. To develop efficient and successful cryopreservation protocols for the widespread applications of hES cells, it is necessary to understand how the conventional protocol (using DMSO as cryoprotectant and a slow freezing rate of 1°C/min, which is effective to protect most mammalian cells), fails to protect the hES cells against damage caused by cryopreservation. It is now clear that low recovery is largely caused by apoptosis, rather than necrosis,^2^ although the details of the mechanisms of apoptosis are still not clear. This is important, as it guides the effort to improve hES cell recovery towards prevention of apoptosis rather than, e.g. optimisation of freezing and thawing protocols to prevent necrosis.^3^

The aim of this study is to identify possible pathways and mechanisms leading to apoptosis following standard cryopreservation. This includes investigating Rho-associated kinase (ROCK) and p53 activation, the effect of ROS and actin dynamics.

Apoptosis (programmed cell death) is regulated by the extrinsic and the intrinsic pathways. In the extrinsic pathway, apoptosis is triggered by the death ligand which activates the death receptor at the cell surface. The death receptor then binds Fas-Associated Death Domain (FADD) in the death-inducing signaling complex (DISC) containing FADD, caspase-8 and caspase-10. In some cells, processed caspase-8 directly initiates the caspase cascade by processing the effector of caspase-3, -6, and -7, then finally leads to apoptosis.^4^ In other cases, activated caspase-8 triggers the intrinsic pathway. This leads to changes in the mitochondrial outer membrane permeability, resulting in the release of cytochrome c into the cytosol.^4^ This is accompanied by the activation of caspase-9, which in turn causes apoptosis. In another intrinsic pathway, signals from external insults can stimulate DNA damage, which can result in the activation of p53. The activation of p53 contributes to the activation of capspase-9 and ultimately apoptosis.^5^

Mammalian cells rapidly produce ROS, including H_2_O_2_, superoxide anion, singlet oxygen, and hydroxyl radical (.OH), in response to various stimuli. Production of ROS plays a vital role in apoptosis through the mitochondrial pathway. Regulation of production and removal of ROS is carried out by antioxidant machinery in cells.^6^ Once the concentration of ROS exceeds the capacity of cellular antioxidant protective mechanisms, it leads to cell damage which cannot be repaired.

In the intrinsic pathway, apoptosis is induced by the activation of p53. Normally p53 is retained in the cytosol at low concentrations and prevented from entering the nucleus. In response to cellular stress, p53 is activated, leading to cell apoptosis either in a transcription-independent manner or a transcription-dependent manner. In the transcription-dependent mechanism, p53 transcriptionally regulates genes which relate to the generation of ROS and trans-activates downstream proapoptosis genes. In the transcription-independent mechanism, p53 induces apoptosis through direct action on mitochondria.^7^, ^8^ p53 binds to the outer mitochondrial membrane, resulting in changes in the permeability of the mitochondrial membrane, then triggers the release of cytochrome c and eventually leads to the activation of caspases. Overall, responses of p53 to ROS insults are dependent on the cell type and the severity of stress.^9^ For example, mouse embryonic stem cells are sensitive to ROS. They are not able to grow without the antioxidant 2-mecaptoethanol (2-ME), likewise hES cells.

The actin cytoskeleton plays an important role in cellular functions such as motility, cell shape and signal-response coupling.^10^ The regulation of actin assembly and disassembly is essential to control the complex signaling systems associated with the external signals to remodeling events leading to alteration of cellular activity to adapt to new living environment.^10^ Apoptosis can be induced by alteration of the equilibrium between G-actin and F-actin inside mammalian cells in response to environmental change.^11^ The equilibrium between G-actin and F-actin is tightly controlled by actin regulatory proteins such as gelsolin, cofilin, coronin, and β-thymosins.^11^

Cytoskeleton dynamics are regulated by the Rho GTPases which help control cellular functions such as cell adhesion, differentiation and proliferation.^12^ The Rho family includes Rho proteins (RhoA and RhoB), Rac1 and Cdc42. One Rho effector, ROCK, has been implicated in the regulation of apoptosis.^13^, ^14^ The ROCK pathway can be activated by a variety of extracellular stimuli, including osmotic stress.^15^ The activation of ROCK results in changes in cell permeability, migration, and apoptosis. Furthermore, it has been found that the activation of ROCK leads to ezrin phosphorylation, resulting in Fas clustering in embryonic fibroblasts and eventually apoptosis through the extrinsic pathway.^16^ As demonstrated in a previous study, the ROCK inhibitor Y-27632, can improve hES recovery during single-cell passaging.^17^ Furthermore, it has been demonstrated that the presence of ROCK inhibitor during freezing and post-thawing can enhance the cell survival rate and colony formation.^18^, ^19^

It is presently unclear why cryopreservation results in such a low recovery rate of hES cells. Low temperature effects on apoptosis is cell-type related.^20^, ^21^ Several studies have noticed the role of caspase proteases in cell death after cryopreservation.^22–25^ It is interesting to note that in some cases, caspases are regarded as a critical element for regulation of cell death.^23^, ^24^ However, inhibition of caspase activity does not necessarily appear to offer a protective effect on cells.^26^ Apart from the contribution of ROCK inhibition to protect apoptosis following the cryopreservation,^18^, ^19^ the possible contribution of other apoptosis mechanisms discussed previously to cryopreservation-induced apoptosis have not yet been identified. In this study, experiments were conducted to identify contributions of these possible mechanisms, to quantify ROS and actin dynamics, and to investigate p53 involvement in cryopreservation-induced apoptosis.

## Materials and Methods

### Maintenance culture of hES cells

The human embryonic stem cell line, HUES2 (Howard Hughes Medical Institute, Department of Molecular and cellular Biology, Harvard University), which was approved by the UK Stem Cell Bank Steering Committee, was used in this study. Maintenance culture of hES cells was carried out by two different culture methods: feeder-dependent culture and feeder-independent culture. In the feeder-dependent culture, hES cells were cultured on a feeder layer of mitomycin C-inactivated mouse embryo fibroblasts (MEF) on 0.1% gelatin-coated plates, in complete hES culture medium containing knockout Dulbecco's modified Eagle's medium, supplemented with 10% KO-Serum Replacement, 1% nonessential amino acids, 2 mM Glutamax-I (all from Invitrogen GIBCO, UK), 0.055 mM β-mercaptoethanol (Sigma-aldrich, UK), and 10 ng/mL basic fibroblast growth factor (bFGF) (R&D systems, UK) at 37°C, under 5% CO_2_ and 21% O_2_. Culture medium was changed 50% daily. For feeder-independent culture, hES cells were cultured on matrigel-coated plates (BD Biosciences, UK) at the dilution factor of 1:100, in MTeSR™ culture medium (Stem Cell Technologies, France), at 37°C, under 5% CO_2_ and 21% O_2_. Culture medium was completely changed daily.

For passaging after 5–7 days of culture, hES colonies were detached by TrypLE™ Express (Invitrogen GIBCO, UK) at 37°C for 5–7 min, followed by gentle flushing by pipette several times to detach hES cells from the feeder layer. Undifferentiated dissociated hES cells were transferred to fresh MEFs plates or matrigel-coated plates which were prepared in advance in the presence of 10 μM ROCK inhibitor Y-27632 (Merck Chemicals, UK) during the first day of culture. The seeding density was 2.5 × 10^4^ cells/cm^2^. The colony efficiency was 1:200.

### Cryopreservation

DMSO (Sigma-Aldrich, UK) was used as the sole CPA in this study. Freezing solution containing 20% of DMSO in the complete hES culture medium or MTeSR culture medium (no extra fetal bovine serum) was made. The cells were detached by TrypLE, centrifuged, and resuspended into the appropriate culture media at a density of 2 × 10^6^ cells/mL. An equal volume of freezing solution was added to the volume of cell suspension, resulting in a final cell density of 1 × 10^6^ cells/mL and 10% (vol) of DMSO. The cells were kept on ice for 30 min to enable DMSO to get into the cells and reach equilibrium. Then the cells were frozen until −80°C using Mr. Frosty (Nalgene, Denmark) in a −80°C freezer. The cells were stored in liquid nitrogen until further use. Before use, the CPA was removed. Briefly, the cells were rapidly thawed in 37°C water bath. Immediately after thawing, the cell suspension was diluted to 10 mL in PBS, centrifuged, and the added DMSO was removed.

### DMSO exposure–DMSO addition and removal without freezing

For DMSO addition only, an equal volume of freezing solution (20% DMSO) was added to the volume of cell suspension at a density of 2 × 10^6^ cells/mL. The cells were kept on ice for 30 min.

For DMSO addition/removal without freezing, as mentioned before, after DMSO addition, the cells were kept on ice for 30 min. Then the cells were diluted to 10 mL of PBS, and the added DMSO was removed by centrifuge.

### Assessment of cell viability immediately after cryopreservation

Cell viability detection immediately after cryopreservation was carried out using Propidium Iodide (PI, BD Bioscience, UK) staining. Following DMSO removal, the cell pellet was resuspended into 1 μM of PI for 5–10 min in the dark. The cells were then washed with PBS to remove extracellular PI. Cell viability was quantified by flow cytometry, using a Becton Dickinson FACsort and CellQuest software, and analysis was carried out with FlowJo software. In each measurement, 20,000 events were measured.

### Assessment of apoptosis 2 h after culture

The combination of Annexin V-FITC (BD Bioscience, UK) and Propidium Iodide was used to identify the stage of apoptosis of the cells. To obtain apoptosis rate for hES cells only, hence, we chose the cells from feeder-independent culture to do apoptosis test. After DMSO was removed, the cell pellet was resuspended into the appropriate culture medium in the absence or the presence of 10 μM Y-27632, and then were plated in 96-well plates coated with matrigel. 2 h later, the cells were collected and washed with cold PBS, resuspended in 1× binding buffer at 1 × 10^6^cells/mL, then stained with Annexin V-FITC and Propidium Iodide at room temperature for 15 min in the dark, and kept on ice before analyzing using flow cytometry. The results were expressed as the percentage of cells experiencing early apoptosis.

### Caspase activity assay

Caspase activities were determined by using Caspase-Glo 8 for caspase-8 and Caspase-Glo 9 caspase-9 assay (Promega, USA). hES cells after regular passage or after cryopreservation procedure were then plated on matrigel-coated 96-well plates in the presence of either 10 μM Y27632 (R), 10 μM Y-27632, and 1μM pifithrin-μ (P),^27^ or 20 μM caspase-9 inhibitor (C9), or 20 μM caspase inhibitor VI (Z), 200 μM Bax-inhibiting peptide (Bax) (all from Merck Chemicals, UK) or in the absence of any inhibitors on the first day of culture, at the density of 7.5 × 10^4^ cells/well. Equal volume of reagent (Caspase-Glo 8 or Caspase-Glo 9) was added to the tested wells after 2 or 24 hr of culture following cryopreservation. After incubation with reagent for 1 hr in the dark at room temperature, caspase-8 and caspase-9 activity were measured using a luminometer (Lucy, UK), respectively.

### Cell recovery rate

In feeder-dependent culture, colonies were clearly determined after 5 days of culture. In contrast, 4 days of culture in feeder-independent culture was long enough to quantify the cell number at the tested condition. Cell seeding density after cryopreservation or after DMSO addition/removal was 1 × 10^5^ cells/cm^2^.

#### Detection of hES Colony Formation from Feeder-Dependent Culture Using Alkaline Phosphatase Staining

Cells after cryopreservation or after DMSO addition/removal were plated on the feeder layer into 48-well plates in the presence of either 10 μM R, or 10 μM R and 1μM P, or 20 μM C9, or 20 μ Z, or 200 μM Bax, or in the absence of any inhibitors on the first day of culture. After additional 4 days of culture in the absence of any inhibitor, cells were washed with PBS twice, stained with 5-bromo-4-chloro-3-indolyl phosphate and nitro blue tetrazolium (BCIP-NBT) (Sigma-Aldrich, UK) at room temperature for at least 2 h in the dark. Images were taken using a Nikon Coolscope (Nikon, Japan). The colony number formed was counted.

#### Cell Proliferation in Feeder-Independent Culture

Cells after cryopreservation or after DMSO addition/removal were plated on matrigel-coated wells of 48-well plates in the presence of either 10 μM R, or 10 μM R and 1μM P, or 20 μM C9, or 20 μ Z, or 200 μM Bax, or in the absence of any inhibitors. After additional 3 days of culture in the absence of any inhibitor, the cells in each well were dissociated using TrypLE, resuspended in 400 μL PBS and the cell number was counted using a disposable cell counting slide (Fastread, immune systems, UK).

### Intracellular ROS

ROS level in the cells from feeder-independent culture after regular passage, after cryopreservation and after DMSO addition/removal was assessed. The cells after treatment were immediately stained with 2′7-Dichlorofluorescein diacetate (DCFH-DA) (Sigma-Aldrich, UK) for hydrogen peroxide or with dihydroethidine (DHE) (Sigma-Aldrich, UK) for superoxide anion generation, for 30 min on ice in the dark. Once DCFH-DA is oxidized to dichlorodihydrofluorescein (DCFH), it becomes fluorescence sensitive at an excitation wavelength of 520 nm and emission wavelength of 610 nm. DHE is excited at 488 nm and emits at 567 nm once it is oxidized. After staining, the cells were washed with PBS and 20,000 cells were analyzed by flow cytometry. Fluorescence intensity was used to indicate intracellular ROS level.

### F-actin assessment

F-actin in cells from feeder-independent culture after regular passage, after cryopreservation, after DMSO addition, and after DMSO addition/removal was quantified as described.^28^ To quantify F-actin, the cells after treatment were fixed in the corresponding solution containing 4% formaldehyde (Sigma-Aldrich, UK) for 15 min, pelleted by centrifuge, permeabilised with 0.1% Triton X-100 (Sigma-Aldrich, UK) in PBS and stained with 0.33 μM rhodamine phalloidin (Invitrogen GIBCO, UK) for 15 min at 4°C, and washed with PBS. F-actin content was quantitatively determined by flow cytometry. Twenty thousand cells were measured at each condition. Fluorescence intensity was used to indicate intracellular F-actin content.

For the detection of F-actin distribution, cells after treatment were fixed with 4% formaldehyde for 15 min, labeled with 0.33 μM rhodamine phalloidin for 15 at 4°C, washed with PBS, transferred to slides, and mounted with the SlowFade kit containing DAPI (Invitrogen GIBCO, UK). Stained cells were visualized by fluorescence microscopy (Nikon, Japan) connected to a cooled charge-coupled device video. Images were collected with ART-1 software.

### p53 assessment

Cells after regular feeder-independent culture and after cryopreservation were fixed with 4% formaldehyde in PBS for 15 min at room temperature, washed 3 times with PBS, incubated with ice-cold 100% methanol (Sigma-Aldrich, UK) for 10 min at −20°C, washed with PBS and blocked with 5% normal donkey serum (Jackson ImmunoResearch, USA) in PBS containing 0.3% Triton-×100 for 60 min. Cells were incubated with the primary antibody, p53 mouse mAb (1:1000) (Cell signaling Technology, UK) which detects and binds to phosphorylated p53, overnight at 4°C, washed with PBS three times and incubated with the secondary antibody, FITC—conjugated donkey anti-mouse IgG (1:100) (Jackson ImmunoResearch, USA), for 2 h at room temperature in the dark. After a final wash with high salt PBS containing 0.4 M NaCl, the cells were transferred to slides, mounted with the SlowFade kit containing DAPI, and visualized by fluorescence microscopy.

### Immunocytochemical characterization of hES cell colonies

Immunocytochemical characterization was determined on the colonies from feeder-independent and feeder-dependent culture at passage 2 after cryopreservation. Immunostaining was performed by using a Human Embryonic Stem Cell Marker kit (R&D Systems). The cells were grown on glass coverslips and after 6 days of culture at passage 2 after cryopreservation were washed with PBS, fixed with 4% formaldehyde (Sigma-Aldrich, UK) in PBS for 20 min at room temperature, washed with PBS, permeabilized and blocked with 0.1% Triton X-100, 1% Bovine Serum Albumin (Sigma-Aldrich, UK) and 10% normal donkey serum in PBS for 45 min at room temperature. For surface markers, no permeabilization was required. Primary antibodies, goat anti-Nanog polyclonal antibody, goat anti-Oct3/4 polyclonal antibody, or mouse anti-SSEA4 monoclonal antibody, were added to the cells at the final concentration of 10 μg/mL in PBS overnight at 4°C. Cells were then washed 3 times with 1% BSA in PBS. Secondary antibodies, FITC-conjugated donkey anti-goat IgG and FITC—conjugated donkey anti-mouse IgG (all from Jackson ImmunoResearch) were diluted at 1:100 in PBS containing 1% BSA. The diluted secondary antibody was applied to the cells for 60 min at room temperature in the dark. Cells were then washed with PBS 3 times, mounted with the SlowFade kit containing DAPI, and visualized by fluorescence microscopy.

### Statistical analysis

Experiments were carried out at least three separate times for each tested condition. Data are presented as means ± sdev of the means for the experiments. The statistical significance was assessed using one-way ANOVA. A probability of *P* < 0.05 was considered to be significant.

## Results

### Cell viability and apoptosis after cryopreservation

Cell viability after cryopreservation was 80.74% ± 2.29% (*n* = 4) and 82.39% ± 2.98% (*n* = 4) for feeder-dependent and feeder-independent culture, respectively. Thirty percent of total population of cryopreserved cells after 2 h of culture were expressed early stage of apoptosis (PI negative and Annexin V positive). The presence of inhibitor either R or the combination of R and P did not affect this early apoptosis.

### Caspase activity

**Figure 1:**
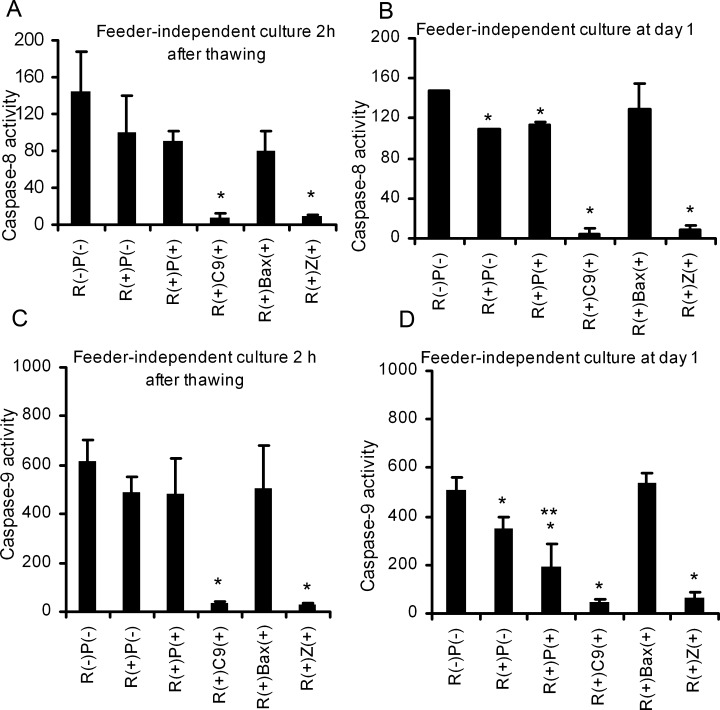
Caspase activity of the cells from feeder-independent after 2h of culture, and at day 1. hES cells after regular passage or after cryopreservation procedure were plated on matrigel-coated 96-well plates in the presence of either 10 μM Y27632, 10 μM Y-27632 (R) and 1 μM pifithrin-μ (P), or 20 μM caspase-9 inhibitor (C9), or 20 μM caspase inhibitor VI (Z), or 200 μM Bax-inhibiting peptide (Bax) or in the absence of any inhibitors on the first day of culture, at the density of 7.5 × 104 cells/well. (A) Caspase-8 activity of the cells cryopreserved by 10% DMSO after 2 h of culture. (B) Caspase-8 activity of the cells cryopreserved by 10% DMSO at day 1 of culture. (C) Caspase-9 activity of the cells cryopreserved by 10% DMSO after 2 h of culture (D) Caspase-9 activity of the cells cryopreserved by 10% at day 1 of culture. Results are expressed as the mean of three independent experiments ± the standard derivation (*n* = 3). Statistical analysis was performed using one-way ANOVA. *P < 0.05, compared with in the absence of any inhibitor. **P < 0.05, compared with in the presence of 10 μM Y-27632 and 1 μM pifithrin-μ.

Caspase activity, an indicator of apoptosis pathways, was investigated during cryopreservation and subsequent culture in this study. As shown in Figure 1, caspase-9 and caspase-8 activity was measured at 2 h at day 0 and at day 1 after plating following cryopreservation. After 2 h of culture, the activity of caspase-9 reached the same level for all tested conditions except when C9 or Z was present in the subsequent culture medium. Caspase-8 activity had a similar trend as caspase-9 at 2 h.

At day 1, compared to the caspase-8 activity without any inhibitors in the subsequent culture, a significant reduction by 20, 60, 90, and 90% was observed when R, or the combination of R and P, or C9, or Z was present in the culture medium. In addition, the combination of R and P resulted in around 30% lower in caspase-8 activity than that in the presence of R alone. Simultaneously, we observed that the presence of R, or the combination of R and P, contributed to the decrease in caspase-9 activity by 20%.

### Cell recovery

**Figure 2:**
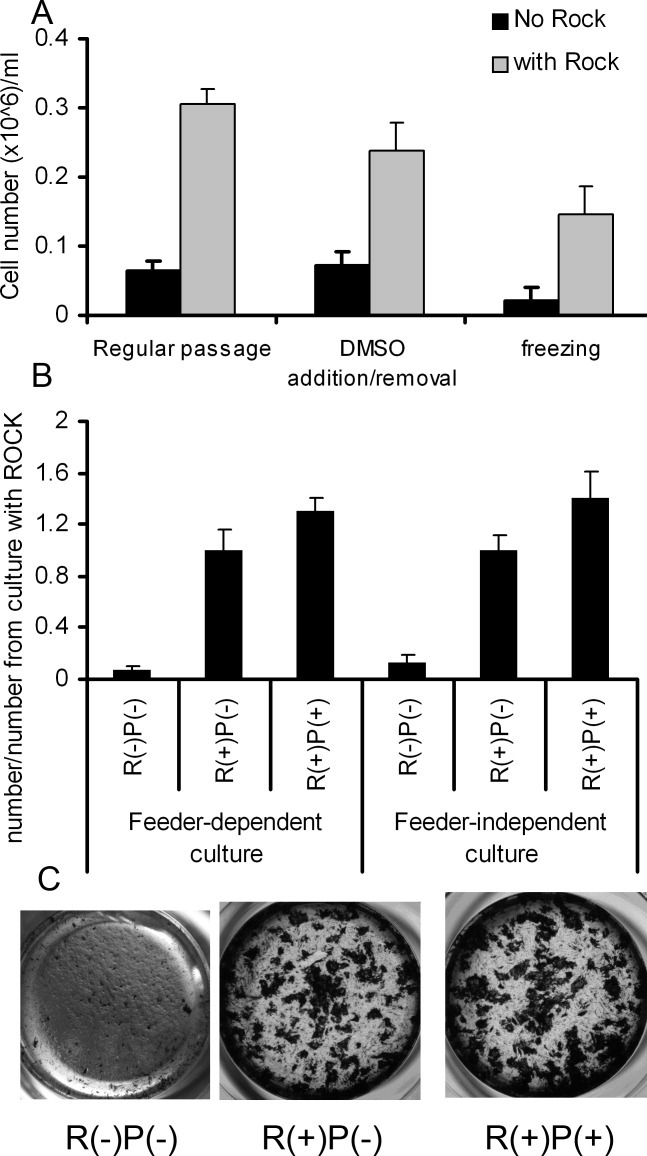
Cell recovery after CPA addition and removal only, and after cryopreservation using 10% DMSO. (A) Cell number per well after 4 days of culture for the cells from feeder-independent culture after regular passage and CPA addition and removal only. (B) The ratio of cell number after cryopreservation, cultured in the absence of Y-27632, in the presence of 10 μM Y-27632, and 10 μM Y-27632 and 1 μM pifithrin-μ to the cell number after cryopreservation, cultured in the presence of Y-27632Y-27632 (+)/(−): R(+)/(−), pifithrin-μ (+)/(−) : P(+)/(−). Results are expressed as the mean of three independent experiments ± the standard derivation (*n* = 4). Statistical analysis was performed using one-way ANOVA. *P < 0.05, compared with regular passage. **P < 0.05, compared with CPA addition and removal only. ***P < 0.05, compared with R(−)P(−). ****P < 0.05, compared with R(+)P(−). (C) Typical colony formation from feeder-dependent culture after cryopreservation using 10% DMSO, and then recovered in the absence of Y-27632, in the presence of 10 μM Y-27632, 1 μM pifithrin-μ and 10 μM Y27632. Scale bar: 100 μm.

To investigate the low recovery rate after cryopreservation, we first detected how DMSO addition/removal without freezing affected the cell recovery rate. As seen in Figure 2A, exposure to DMSO did not affect cell recovery during regular passaging with or without ROCK inhibitor.

Figure 2B summarizes the results of cell recovery following cryopreservation, when the cells were cultured in the medium supplemented with either R, the combination of R and P, or without any inhibitors during the 1st day of culture. For both feeder-dependent and feeder-independent culture, the presence of R significantly improved the cell recovery rate after cryopreservation. The combination of R and P presented in the subsequent culture medium further significantly enhanced the cell recovery rate, as shown in Figure 2B.

Typical colony formation after cryopreservation is shown in Figure 2C. In the absence of any inhibitors, it was hard to detect any colonies after 5 days of culture. In contrast, more, larger colonies were observed in the presence of either R or the combination of R and P.

**Figure 3:**
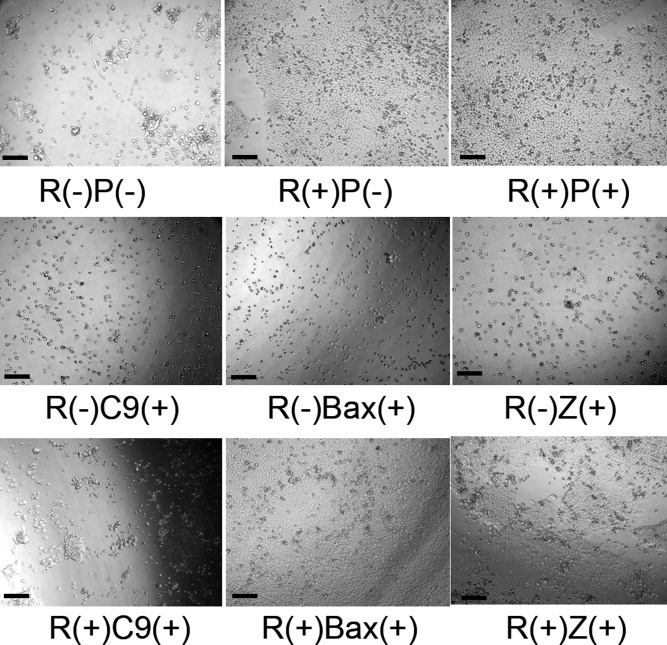
Colony formation from feeder-independent culture after cryopreservation using 10% DMSO, and then recovered in the absence of Y-27632 [R(−)P(−)], in the presence of 10 μM Y-27632 and 1 μM pifithrin-μ [R(+)P(−)], 20 μM caspase-9 inhibitor [R(−)C9(+)] , 20 μM -VAD-FMK [R(−)Z(+)], 200 μM Bax-inhibiting peptide [R(−)Bax(+)], or in the combination of Y-27632 and 20 μM caspase-9 inhibitor [R(+)C9(+)], 20 μM -VAD-FMK [R(+)Z(+)], 200 μM Bax-inhibiting peptide [R(+)Bax(+)], respectively. Scale bar: 50 μm.

Figure 3 shows how the inhibitors C9, Z and Bax affected the cell recovery after cryopreservation. As seen in Figure 3, when only C9 or Z inhibitor was presented in the subsequent culture medium, it was hard to detect any colonies, similar to that when no inhibitor was present. Cell recovery was greatly increased by the combination of R with C9, Bax or Z, but was still much less than R only or the combination of R and P.

### Intracellular ROS

**Figure 4:**
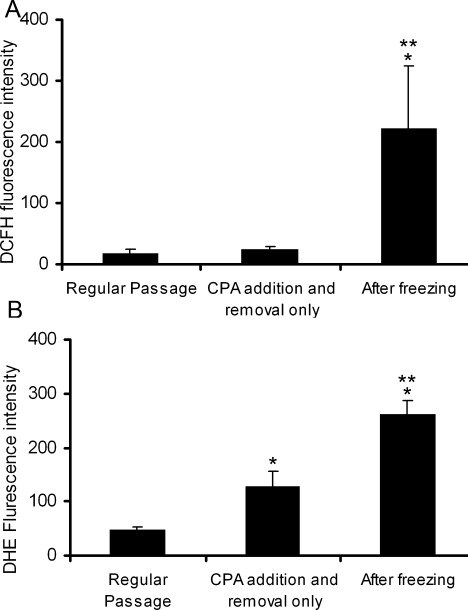
Intracellular ROS after regular passage, CPA addition and removal only, and after cryopreservation. The cells were stained with 2′7-Dichlorofluorescein diacetate (DCFH-DA), for hydrogen peroxide, or with dihydroethidine (DHE) for superoxide anion generation. (A) Hydrogen peroxide in mitochondria. (B) Superoxide content in mitochondria. Results are expressed as the mean of three independent experiments ± the standard derivation (*n* = 4). Statistical analysis was performed using one-way ANOVA. *P < 0.05, compared with regular passage. **P < 0.05, compared with CPA addition and removal only.

Cryopreservation could cause damage to mitochondria, hence the freezing effects on ROS generation on mitochondria were studied. The level of superoxide anion generation and hydrogen peroxide in the cells after regular passage, after DMSO addition/removal, and after cryopreservation was quantified. The level of superoxide anion and hydrogen peroxide in the cells was measured by looking at the DCFH and DHE intensity. As seen in Figure 4, a dramatic increase in hydrogen peroxide occurred with freezing compared to regular passage. The DCFH intensity increased by nearly 10-fold after cryopreservation compared to regular passage. Simple exposure of the cells to DMSO and following removal per se did not affect the concentration of hydrogen peroxide, so the effect can be attributed to the freezing procedure rather than the cryoprotectant.

The level of superoxide anion concentration, measured by the DHE intensity, increased around 2-fold after DMSO addition/removal and 5-fold after cryopreservation.

### Intracellular F-actin

**Figure 5:**
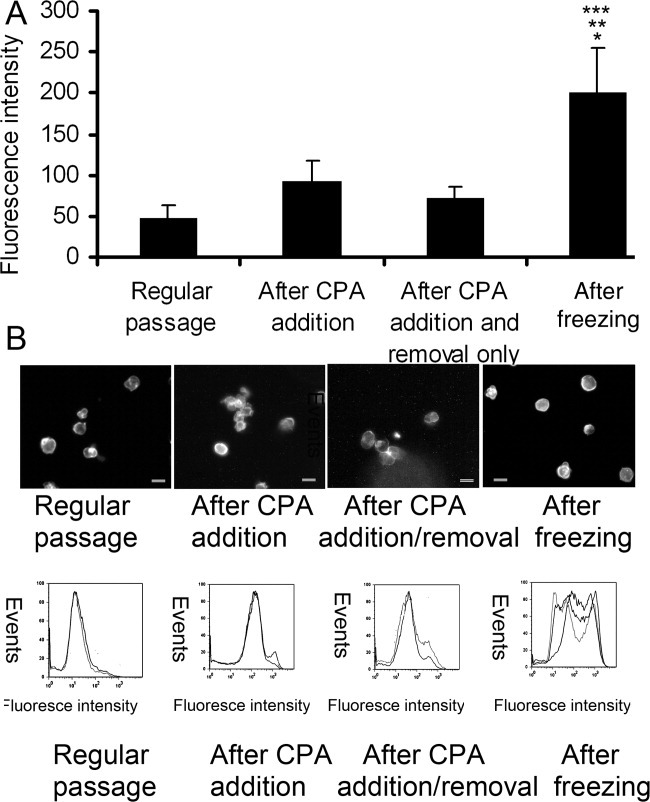
Distribution and quantification of intracellular F-actin after regular passage, CPA addition only, CPA addition and removal only, and cryopreservation by 10% DMSO. To quantify F-actin, the cells after treatment were fixed in the corresponding solution containing 4% formaldehyde, permeabilised with 0.1% Triton in PBS, and stained with 0.33 μM rhodamine phalloidin. F-actin content was quantitatively determined by flow cytometry. For the detection of F-actin distribution, the cells labeled with 0.33 μM rhodamine phalloidin were transferred to slides, and visualized by fluorescence microscopy. (A) intracellular F-actin content after regular passage, CPA addition only, CPA addition and removal only, and cryopreservation by 10% DMSO. Results are expressed as the mean of three independent experiments ± the standard derivation (*n* = 3). Statistical analysis was performed using one-way or two factor with replication method. *P < 0.05, compared with regular passage. **P < 0.05, compared with after CPA addition, ***P < 0.05, compared with CPA addition and removal only. (B) F-actin distribution in suspension of hES after regular passage, CPA addition only, CPA addition and removal only, and cryopreservation by 10% DMSO. Scale bar: 10 μm. (C) Representative FAC analysis.

The cytoskeleton is one of the most important parameters of cell function. The amount of F-actin in the cytoskeleton could affect cell signaling pathways, and thereby cell functions. Hence, the amount of intracellular F-actin was measured after regular passage, DMSO addition, DMSO addition/removal, and after cryopreservation. As shown in Figure 5, DMSO addition significantly increased F-actin content by around 2-fold, compared to regular passage. A further accumulation in F-actin content, 4-fold, occurred after cryopreservation, compared to regular passage.

The distribution of F-actin inside the cells can regulate cell function. As shown in Figure 5, staining was pale and without a clear ring after regular passage. Exposure of the cells to DMSO resulted in an increase in F-actin, and this strong color spread across the cells. In contrast, after DMSO addition/removal, F-actin was distributed more like that after regular passage. Importantly, after cryopreservation, F-actin did not distribute uniformly across the cells. Instead F-actin accumulated at the edge of the cells. A clear ring around the cells and membrane blebbing was observed.

### p53 assessment

**Figure 6:**
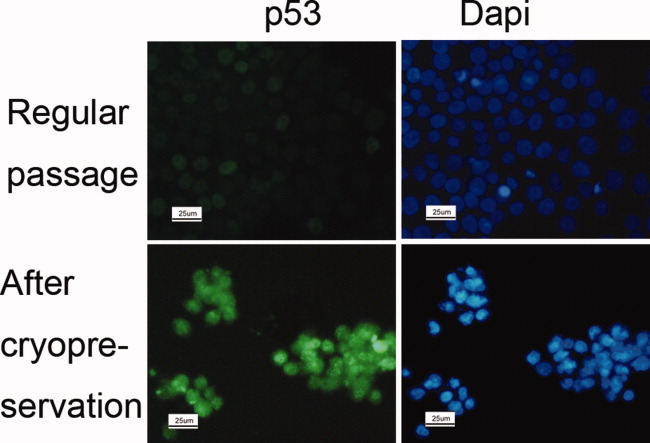
p53 expression of the hESs after cryopreservation using 10% DMSO. p53 was expressed in green color, and blue color was for DAPI. Scale bar: 25 μm.

Figure 6 shows p53 expression in hES cells after cryopreservation. In cells with normal culture, p53 was expressed in very few cells. In contrast, we observed that the cryopreservation procedure resulted in strong p53 expression in the nucleus of the cells.

### Immunocytochemical characterization

**Figure 7:**
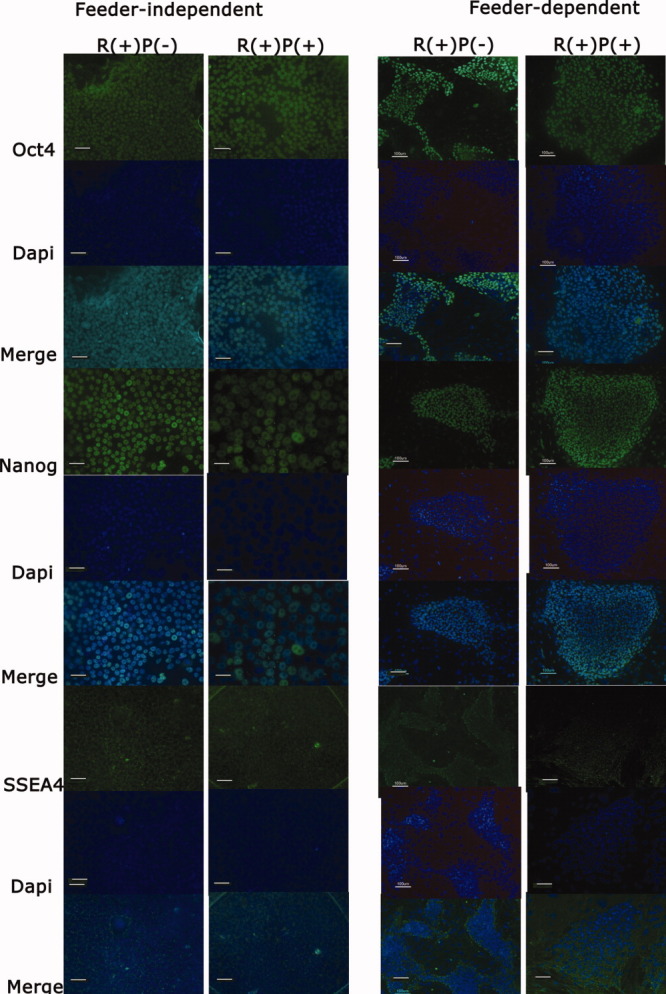
Expression of undifferendiated marker, nuclear markers, OCT4 and Nanog, and surface marker, SSEA4, for hES cells at passage 2 after cryopreservation using 10% DMSO. The cells after cryopreservation were cultured in the presence of ROCK inhibitor, R(+)P(−), or in the presence of ROCK inhibitor and pifithrin-μ, R(+)P(+). Scale bar: 50 μm for feeder-dependent culture and 25 μm fro feeder-independent culture, respectively.

The maintenance of undifferentiated phenotype of the hES cells after cryopreservation was assessed by October 4, Nanog and SSEA4, all markers of an undifferentiated phenotype, at passage 2 after cryopreservation from feeder-dependent and feeder-independent culture. Typical images are shown in Figure 7. The cells from feeder-independent and feeder-dependent culture maintained their undifferentiated state following cryopreservation and recovery.

## Discussion

The obstacle faced, using slow-freezing with 10% DMSO as a cryoprotectant for hES cells, is an extremely low cell survival rate, around 10%.^2^ It has been demonstrated by Heng et al.^2^ that the low cell recovery rate after conventional cryopreservation is associated with apoptosis rather than necrosis induced by cryoinjury, which is consistent with our observations. However, the mechanism behind the induction of apoptosis by cryopreservation is still not clear. Based on results presented here, we propose that ROS production, ROCK activation, change in F-actin and activation of p53 work together as a network and contribute to the induction of apoptosis in cryopreservation, as proposed in Figure 8.

**8:**
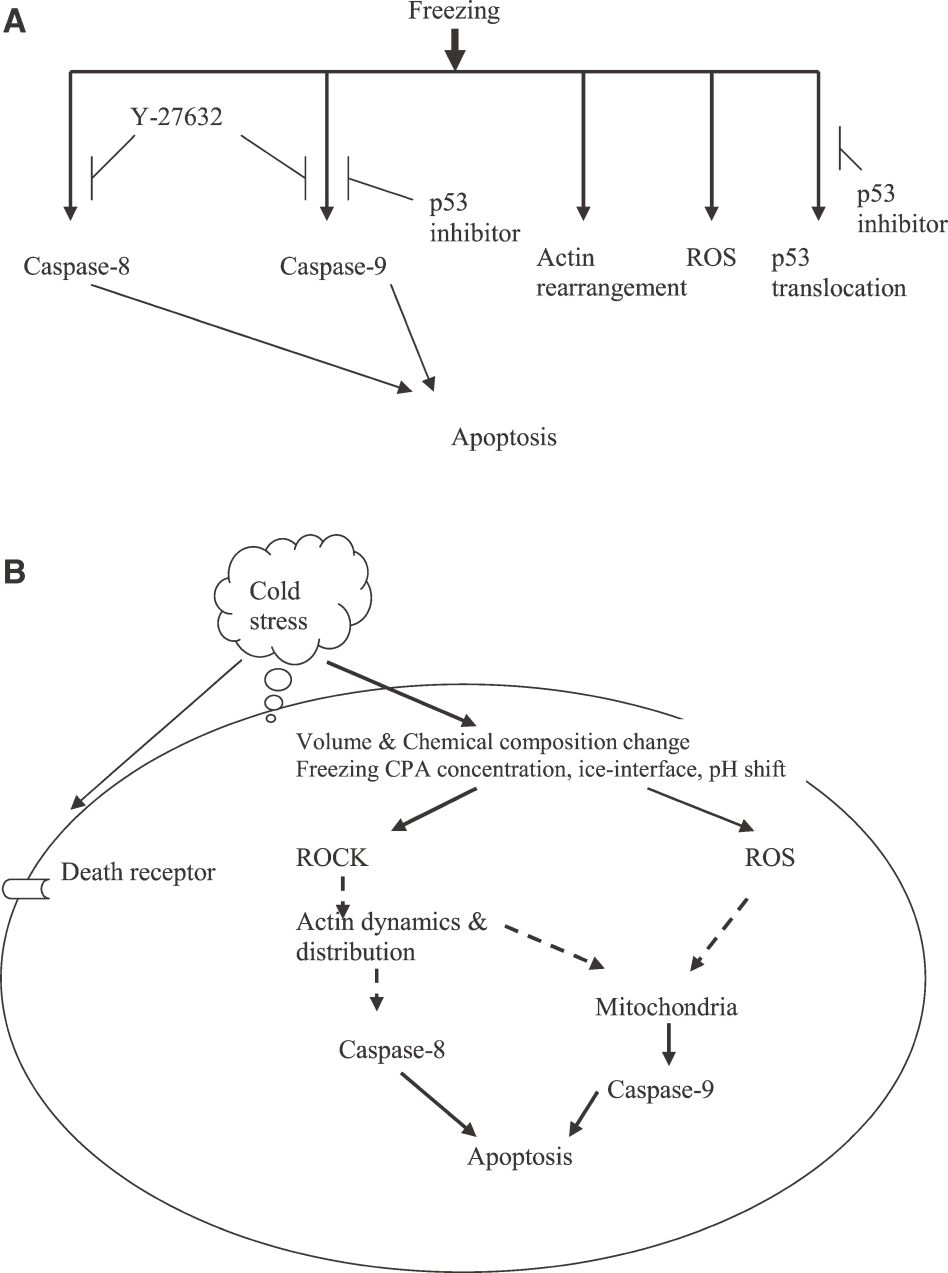
A) Observed mediators of apoptosis induced during cryopreservation; (B) Suggested mechanism of cell responses to cryopreservation.

In cryopreservation, cells experience osmotic stress caused by osmolarity differences between the intracellular and extracellular environment during DMSO addition, DMSO removal, and the procedure of freezing. Osmotic shock leads to cell volume change due to the semi-permeability of the cell membrane.^29^ Cell volume change further results in chemical composition change inside cells. Moreover, during freezing in cryopreservation, apart from osmotic stress, cells are exposed to cold stress such as freezing concentration, ice-interface, precipitation of salts and pH shift. The present study has shown that the low cell recovery rate is mainly caused by freezing in cryopreservation, rather than by the procedure of DMSO addition/ removal (Figure 2A). It suggests that cold stress and osmotic stress during freezing together mainly contributes to induction of apoptosis, and further leads to low cell recovery.

The intracellular level of ROS correlates with apoptosis.^30^ Once it exceeds the capacity of cellular antioxidant protective mechanisms, it results in cell death. The rise in ROS mainly results from the procedure of freezing in cryopreservation, as the level of superoxide anion and hydrogen peroxide after cryopreservation was significantly greater than that in the regular culture or after DMSO addition/removal (Figure 4). Hence, the cryopreservation procedure leaves to the cells exposed to oxidative stress. The production of superoxide anion induces the release of cytochrome c from the intermembrane of mitochondria to cytosol.^31^ This facilitates the activation of a multiprotein complex including Apaf-1, cytochrome c, and caspase-9 and eventually leading to apoptosis.^32^ Hence, intracellular ROS suppression is a potential route to improve the cell recovery after cryopreservation.

p53 plays a critical role in regulation of apoptosis in response to cellular stimuli.^33^ It has been demonstrated that p53 acts as a survival factor at low levels of ROS, but induces pro-oxidant genes at high concentrations of ROS.^9^ Correlating with the high level of intracellular ROS caused by the procedure of freezing in cryopreservation, p53 was activated, and accumulated in the nucleus after cryopreservation (Figure 6). The presence of p53 inhibitor in the subsequent culture medium resulted in the reduction of caspase-9 activity (Figure 1D), but not caspase-8 activity. This reduction suggests that apoptosis after cryopreservation is partly induced by the activation of p53 through the intrinsic pathway. In hES cells, p53 has been shown not to be activated by the transcription-dependent pathway.^34^ Thus, under stress caused by cryopreservation, the activation of p53 probably triggers apoptosis through the mitochondrial pathway.

To suppress apoptosis induced by cryopreservation, we need to identify whether apoptosis is caused by the extrinsic or intrinsic pathway. Our finding is consistent with a previous study, in which hyperosmotic stress stimulates the death receptor^35^, ^36^ and eventually leads to activation of caspase-8^37^ (Figure 1). We have shown that inhibition of ROCK following cryopreservation results in the reduction of caspase-8 activity (Figure 1D), therefore activation of ROCK (presumably promoting apoptosis through ezrin phosphorylation and then in turn leading to apoptosis through the extrinsic pathway^16^) is involved in the regulation of activation of caspase-8 in cryopreservation. However, we cannot identify whether the activation of caspase-8 directly induces apoptosis, or indirectly through the mitochondrial pathway. Further study is needed to dissect the pathway further.

As both caspase-8 and caspase-9 are activated in cryopreservation, we tested whether inhibition of caspase activity can suppress apoptosis and improve the cell recovery. The presence of C9 or Z,significantly inhibits caspase-8 and caspase-9 activity (Figure 2). However, it is interesting to note that the presence of the C9 or Z in the subsequent culture medium cannot enhance the cell recovery after cryopreservation. This is consistent with a previous study showing that inhibition of caspase activity does not appear to offer a protective effect on cells.^26^ It implicates caspase-8 and caspase-9 as downstream effectors in cryopreservation-induced apoptosis. Simply inhibiting downstream caspase activity does not improve cell recovery. It may also indicate that Y-27632 inducing cell recovery is independent of caspase activity. A further investigation is needed to be utilized. Nevertheless, we discovered that Bax-peptide inhibitor can promote the cell recovery to some extent, although not nearly as well as ROCK inhibitor or the combination of ROCK and p53 inhibitors. However, we cannot explain why the presence of C9 or Z completely inhibits caspase-8 activity. The decrease in cell recovery by the addition of C9 and Z may be caused by the inappropriate concentration of C9 and Z.

Hypertonic conditions can affect the cytoskeleton, the remodeling of actin,^38^ and in turn modulate apoptosis signaling.^39^ In cryopreservation, the cells are exposed to anisotonic conditions. This results in elevation of F-actin level in hES cells (Figure 5), in agreement with a previous study.^35^ This elevation could be a major reason for the low cell recovery after cryopreservation, as the alteration in F-actin itself in some cases can induce apoptosis.^38^ It should be noted that F-actin returns to normal levels when DMSO is removed directly from the cells without freezing. This could explain why the procedure of DMSO addition/removal without freezing does not have a detrimental effect on cell recovery. In addition, actin dynamics and redistribution of F-actin can be modulated by effectors of the Rho family (ROCK1 and ROCK2), major regulators of the actin cytoskeleton^13^, ^14^ under osmotic stress during DMSO addition, removal and freezing. The link between F-actin and ROCK activity is needed to be further studied.

We have demonstrated that the presence of ROCK inhibitor in the subsequent culture suppresses apoptosis, consistent with previous studies.^11^, ^12^ We further examined the caspase-8 and caspase-9 activity of hES cells when ROCK inhibitor was present in the subsequent culture after cryopreservation. We noticed that both caspase-8 and caspase-9 activity were decreased due to the presence of ROCK inhibitor (Figure 1). Based on our results, we suggest that the presence of ROCK inhibitor helps to suppress apoptosis and enhance the cell recovery through the inhibition of both the extrinsic and intrinsic pathways.

In addition, the distribution of F-actin within cells intervenes with other stimuli, and further influences cell function.^15^ Under hyper- or hypo-tonic conditions during cryopreservation, remodeling of F-actin occurs. Accompanying the changes in F-actin content during cryopreservation, changes in the distribution of F-actin occurred within hES cells, consistent with observations by Rizoli et al.^38^ How the rigid submembraneous F-actin affects the function of hES cells needs to be elucidated.

## Conclusions

We investigated how the stimuli from each cryopreservation step results in apoptosis, which leads to the low cell recovery rate of hES cells after cryopreservation. The largest effect of cryopreservation, particularly the freezing step, was found to be the sharp increase in the level of ROS in hES. This presumably leads to the activation and translocation of p53, which in turn results in the activation of caspase-9, and ultimately apoptosis. Elevated levels of F-actin during freezing could result in change in apoptosis signals. The inhibition of caspase-8 and caspase-9 activity through ROCK inhibitor suggests that apoptosis is induced through both the intrinsic and extrinsic pathways. However, the inhibition pathway was not identified and needs to be further elucidated. A remaining question is why hES cells are particularly sensitive to cryopreservation, while many mammalian cells, including mouse ES cell survive. Furthermore, other pathways apart from apoptosis as likely causes of the low recovery rates observed for cryopreserved hES cells should be considered.
